# 

**Published:** 1902-01

**Authors:** 


### ILLUSTRATIONS.

PAGE

American Veterinary Medical Association Building, where Clinics were Held September, 1902 . . . . . . . . . 655
Calculi . . . . . . . . . . . . . . . . 273
Canine Instrument, a New . . . . . . . . . . . 235
Canine and Feline Instrument, a New . . . . . . . . 293
Chronic Galactophoritis in the Bitch . . . . . . . . . 575
Contrivance for the Ready Handling of Disabled Horses, a . . . 26
Entero-enteral Anastomosis in the Dog, a Simple Method of Performing . . . . . . . . . . . . . . . 160
Neoplasia Exhibiting Progressive Malignancy, a Condition of . . 292
Pathogenesis of Roaring, Investigation of the—
471, 472, 473, 474, 475, 476, 477, 479, 480, 481
Rayner, Dr. Thomas B. . . . . . . . . . . . 405
Rayner, Moncure Robinson, Deceased . . . . . . . . 405
Some Experiments upon the Immunization of Cattle against Tuberculosis . . . . . . . . . . . . . . . 684–688
Tumor-like Lesion in the Lung of a Horse Caused by a Blastomyces (Torula) . . . . . . . . . . . . . . . 743
Veterinary Department, State College, St. Anthony Park, Minnesota . 654
X-ray as an Aid in the Diagnosis of Tuberculosis in Cattle, the—
98, 99, 100, 101, 102, 103

### ASSOCIATION PROCEEDINGS.

### PENNSYLVANIA STATE VETERINARY MEDICAL ASSOCIATION.

SECRETARY RANCK, of the Keystone State Association, is out with a preliminary notice for the coming annual meeting of this organization, urging all veterinarians of that State to plan attendance and to prepare papers, reports of cases, and other matters of interest to the profession. The meeting on March 4th and 5th promises to be one of unusual interest, and we urge every JOURNAL reader to avail himself of meeting with his Pennsylvania colleagues at least once. The annual meeting is held in Philadelphia. Secretary Ranck is ever ready to answer all questions and afford any information to any and all members of the Association and profession.

### PERSONALS.

Among the first-day visitors to the Dog Show in Philadelphia was Dr. H. Clay Glover, of New York City.

Dr. W. F. Heyde, of St. Louis, Mo., conducts a well-equipped infirmary in connection with his practice.

Dr. C. J. Marshall, of Philadelphia, was veterinarian to the Philadelphia Dog Show in November.

Dr. John E. Brown, Secretary of the Iowa State Veterinary Medical Association, has removed to Chattanooga, Tennessee, owing to the impaired health of members of his family.

Dr. O. M. Norton, graduate of the Veterinary Department of the University of Pennsylvania, and located at Aldenville, Pa., has just returned from a trip to South Africa with a British transport carrying horses and mules.

Dr. W. A. Thomas, of Lincoln, Nebraska, a graduate of the Veterinary Department of Iowa Agricultural College, fills the rôle of State Veterinarian of that State.

Dr. W. B. E. Miller was seriously injured in December by an unprovoked assault by a ruffian in the city of Camden.

Dr. Leonard Pearson, of Philadelphia, Pa., spent several days in New York State during December.

Dr. J. E. Foster, of Coshocton, Ohio, takes an active interest in the general work of the Ohio Society for the Prevention of Tuberculosis.

Governor William A. Stone, of Pennsylvania, has just reappointed Dr. W. Horace Hoskins, of Philadelphia, a member of the State Veterinary Board of Veterinary Medical Examiners.

Dr. T. A. Donaldson, of Manchester, Connecticut, has removed to Hartford, Connecticut, where he will resume active practice again. Dr. Donaldson has quite recovered from his many months of impaired health.

Dr. William Dougherty, of Baltimore, Md., was a visitor to Philadelphia in December to pay a last tribute to the late Dr. Rush Shippen Huidekoper.

Dr. D. E. Salmon, of the Bureau of Animal Industry, has just issued a reprint of an article prepared by him for the *Medical Record*, entitled "Is Rabies a Specific Disease?" Dr. Salmon is one of the foremost and most prolific writers on this subject in the world.

Dr. Lemuel Pope, of Portsmouth, N. H., is to give a course of lectures in veterinary science to the students of the New Hampshire Agricultural College.

Dr. Charles L. Colton, of Englewood, N. J., has associated himself with Dr. R. P. Lyman, of Hartford, Conn., as assistant.

Dr. W. H. Dalrymple, of Baton Rouge, La., has been selected as a member of the Executive Committee of the National Live-Stock Association. Dr. Dalrymple's long years of earnest work in the betterment of live-stock conditions in the South makes his appointment one of special merit.

Dr. Coleman Nockolds, formerly of the Grand Rapids Veterinary College, is now located at Batangos, P. I., with the cavalry regiment to which he is attached.

Dr. Lemuel Pope, of Portsmouth, N. H., was recently operated upon for appendicitis, and is recovering. He had been ill since November 3d, but has now resumed his practice.

Dr. Ora W. Everly, of Holmesville, Ohio, has been appointed assistant inspector of the Bureau of Animal Industry, and has been assigned to South St. Joseph, Mo.

*The Alumni Register* of the University of Pennsylvania, refers to our esteemed colleague, Dr. W. H. Ridge, of Trevose, Pa., as a "scientific tiller of the soil."

Dr. C. Edward Leech, formerly of Milwaukee, Wis., has located at Winona, Minn., where he is actively engaged in practice.

Dr. George W. Pope, Secretary of the Veterinary Medical Association of New Jersey, new address is Athenia, N. J., in place of Garfield. The new quarantine station is at Athenia.

Dr. Veranus A. Moore, of Ithaca, N. Y., was a visitor to Washington, D. C., and Philadelphia early in November.

Dr. Jacob Smith, formerly practising at Gettysburg, Ohio, has removed to Troy, Ohio.

Dr. Leonard Pearson, of Philadelphia, made a flying trip in November through parts of New York, Pennsylvania, Maryland, and West Virginia.

Dr. William Dougherty, of Baltimore, Md., was a visitor to the JOURNAL office in November.

Mr. R. A. Pearson, of the Dairy Department of the Department of Agriculture, was one of the speakers at the State Dairymen's Association in Nebraska in November.

Dr. Leonard Pearson, State Veterinarian, was a visitor to the "Smoky City" in November.

Dr. E. C. Lacock, of Allegheny, Pa., President of the Allegheny County Veterinary Medical Association, was a visitor to Philadelphia early in November.

Dr. P. L. Gaunt, of Burlington, N. J., came near losing his life in November from an accidental dose of aconite taken in mistake.

Dr. A. T. Sellers, of Camden, N. J., was quite painfully injured by the kick of a horse in the sales-mart of West Philadelphia.

Dr. James W. Sallade, of Auburn, Pa., was a visitor to Philadelphia in December. Dr. Sallade is President of the State Board of Veterinary Medical Examiners.

Dr. Raymond Barber, of Newtown, Pa., is a matriculate at the Jefferson Medical College, pursuing a course of human medicine.

Dr. A. T. Sellers, of Camden, N. J., received the appointment of veterinarian to the police department stables in January.

Dr. John W. Adams, of Philadelphia, escaped with a few bruises, in a serious runaway accident in January. Several persons were injured seriously before the death of the mare occurred in her wild flight.

Dr. Charles E. Cotton, of Minneapolis, Minn., spent two weeks in the East during the month of January.

